# Loneliness among community-dwelling older adults across biological, clinical and psychosocial domains before and during the COVID-19 pandemic: a systematic review

**DOI:** 10.3389/fpubh.2026.1831731

**Published:** 2026-08-03

**Authors:** Georgia Casanova, Giorgia Fattorini, Sara Santini, Marta Balietti, Ilaria Barboni, Robertina Giacconi, Giovanni Lamura

**Affiliations:** 1 Centre for Socio-Economic Research on Aging, IRCCS INRCA, Ancona, Italy; 2 Department of Experimental and Clinical Medicine, Section of Neuroscience and Cell Biology, Università Politecnica delle Marche, Ancona, Italy; 3 Center for Neurobiology of Aging, IRCCS INRCA, Ancona, Italy; 4 Clinical Unit of Physical Rehabilitation, IRCCS INRCA, Ancona, Italy; 5 Advanced Technology Center for Aging Research and Geriatric Mouse Clinic, IRCCS INRCA, Ancona, Italy

**Keywords:** biological clinical and psychosocial domains people, community-dwelling, COVID-19, loneliness, multidisciplinary research, older adults, systematic review

## Abstract

**Background:**

Loneliness is a subjective and distressing experience resulting from a discrepancy between desired and actual social relationships and represents an important public health concern among community-dwelling older adults. Although it can occur throughout the life course, older adults are particularly vulnerable because of age-related conditions such as multimorbidity, mobility limitations, sensory impairments, widowhood, and retirement. These factors highlight the need to understand loneliness as a multifactorial phenomenon requiring a multidimensional perspective considering biological, clinical, and psychosocial determinants and consequences.

**Objectives:**

This systematic literature review aims to investigate the extent to which the available literature on loneliness in older age adopts a multidisciplinary approach.

**Research design and methods:**

Multiple databases were searched and papers in biological, clinical and socio-psychological areas were selected according to predefined inclusion criteria and the PRISMA guidelines. Study characteristics (author, year of publication, methodology, and sample size) and contents (keywords, loneliness as primary or secondary outcome, multidisciplinary) were analysed.

**Results:**

One hundred thirty-one articles were selected: 22 biological; 51 clinical and 58 socio-psychological. Eighty-nine studies adopted quantitative methods, mostly concentrated in the biological area, while qualitative and mixed-method approaches are more common in the other two areas. In biological papers, loneliness is treated as an etiopathological factor or pathological condition, thus underestimating or denying its social significance. Clinical papers often associate loneliness with depression and socio-psychological ones do not investigate loneliness biological and clinical implications.

**Discussion and conclusion:**

Although the COVID-19 was associated with a growing number of multidisciplinary studies, research on loneliness among older adults remains largely fragmented across disciplinary boundaries. Strengthening collaboration across biological, clinical, and psychosocial domains could contribute to a more comprehensive understanding of loneliness and support the development of more effective prevention and intervention strategies.

## 1 Introduction

Loneliness is commonly defined as the negative feeling arising from a discrepancy between the desired and the actual quality or quantity of social relationships (1). From this perspective, loneliness represents a subjective and distressing experience resulting from unmet social needs (2) and shows only a weak association with the objective size of one’s social network or the frequency of social interactions (3). Although loneliness and social isolation are often used interchangeably, they represent distinct concepts. Loneliness refers to the subjective perception of inadequate social relationships, whereas social isolation reflects the objective absence or scarcity of social contacts. Consequently, individuals may experience loneliness despite having frequent social interactions, while socially isolated individuals do not necessarily perceive themselves as lonely. Accordingly, the perceived quality and satisfaction of relationships play a crucial role in shaping the experience of loneliness (4). The present review focuses specifically on community-dwelling older adults, defined as individuals living independently in the community rather than in institutional care settings.

Although loneliness does not increase linearly with age, older adults often face circumstances that may increase their vulnerability to loneliness, including declining health, reduced mobility, sensory impairments, living alone, widowhood, or retirement (5, 6). These conditions are more common among individuals aged 80 and over, making this group particularly vulnerable compared to other age cohorts (7).

Given global demographic trends and increasing longevity, loneliness is expected to affect a growing proportion of the older population. Several countries—including the United Kingdom, the Netherlands, and Japan—have recognised its societal relevance by launching policy initiatives, establishing dedicated governmental structures, or appointing political figures responsible for addressing the issue (8, 9). More recently, the COVID-19 pandemic brought unprecedented attention to loneliness, particularly because social distancing measures highlighted its implications for older adults’ health and well-being.

Between 2016 and 2020, loneliness among European citizens doubled to 25%, with the proportion of older adults reporting loneliness increasing from 15 to 23% (10). A growing body of research has therefore examined the effects of pandemic-related restrictions on social isolation and emotional well-being among older populations (11). Beyond increasing the visibility of loneliness, the pandemic also highlighted the complexity of older adults’ health needs and the limitations of fragmented responses, reinforcing the importance of integrated perspectives that combine medical, psychological, and social dimensions of care (12–14).

A substantial part of scholarly attention to loneliness derives from its well-documented consequences for health and well-being. Loneliness has been associated with lower quality of life and higher mortality (15–17), increased risks of cardiovascular and cerebrovascular diseases (18–20), inflammatory processes (21), impaired immune functioning (22–24), cognitive decline and dementia (25, 26), and physical frailty (27). Evidence also suggests a causal relationship between loneliness and stress (28). In older adults, stress may stem from multiple sources, including chronic health conditions, disability, bereavement, shrinking social networks, or financial constraints. Persistent stress responses may dysregulate the hypothalamic–pituitary–adrenal (HPA) axis, weakening immune functioning and increasing vulnerability to disease (28).

For this reason, loneliness among older adults is increasingly conceptualised as a complex health phenomenon that cannot be adequately understood within a single disciplinary framework (25). Taken together, these findings suggest that loneliness among older adults should be understood as a multifactorial condition shaped by the interaction of biological, clinical, and socio-psychological determinants, highlighting the need for perspectives that extend beyond single disciplinary approaches (29).

Addressing such complexity requires collaboration across different fields of research. Collaboration between disciplines lies at the core of both multidisciplinary and interdisciplinary approaches. The key distinction between these perspectives concerns the degree of integration. Multidisciplinary models combine insights from different disciplines while preserving their conceptual and methodological boundaries, allowing complex phenomena to be examined from complementary perspectives (30). Such approaches can support the development of more comprehensive intervention strategies that address biological mechanisms, clinical conditions, and social or relational factors influencing loneliness.

Interdisciplinary approaches involve a deeper integration of knowledge, where disciplinary boundaries are bridged in order to co-produce shared conceptual frameworks (31). More recently, the concept of transdisciplinarity has been introduced to describe collaborative processes that extend beyond academia and involve stakeholders in addressing complex societal challenges (32). The COVID-19 emergency further underscored the relevance of collaborative approaches in the care of older adults, revealing the limitations of fragmented responses to complex health conditions (32).

Accordingly, the present review adopts a multidisciplinary perspective to examine the extent to which biological, clinical, and psychosocial approaches coexist within the literature and collectively contribute to a broader understanding of loneliness among older adults.

While interdisciplinary and transdisciplinary approaches offer highly integrative perspectives, they also require complex organisational and methodological arrangements. In this context, the present review adopts a multidisciplinary analytical framework structured around three major research domains—biological, clinical, and psychosocial—which are widely recognised as central domains in the study of health and ageing.

Although the present review distinguishes biological, clinical, and psychosocial research as separate analytical domains, this classification is conceptually grounded in the biopsychosocial model proposed by Engel (33), which provides the overarching framework linking these complementary perspectives. In gerontological research, this model provides a useful conceptual basis for examining complex phenomena such as loneliness through multiple complementary perspectives (34, 35).

Within this framework, the biological perspective focuses on physiological pathways through which loneliness may influence health, including neuroendocrine regulation, inflammatory responses, and immune functioning (36, 37). The clinical perspective examines the implications of loneliness for health outcomes and disease trajectories, including functional decline, cognitive impairment, depression, and increased mortality risk (38, 39). The psychosocial perspective conceptualises loneliness as a subjective experience embedded in social relationships, social networks, and broader contextual determinants of health, emphasising the role of social integration, perceived support, and community participation (40, 41). Together, these perspectives offer a comprehensive framework for analysing loneliness among older adults.

Despite the expanding literature on loneliness among older adults, limited attention has been paid to how different disciplinary perspectives contribute to its investigation and to the extent to which research in this field adopts a genuinely multidisciplinary orientation. The COVID-19 pandemic represents a particularly relevant context in which to explore these dynamics. By comparing studies conducted before and during the pandemic, it becomes possible to examine whether this global health crisis contributed to greater interaction between biological, clinical, and psychosocial perspectives in the study of loneliness.

Therefore, this systematic literature review aims to assess the extent to which research on loneliness in older adults adopts a multidisciplinary perspective. Specifically, it examines how biological, clinical, and psychosocial studies conceptualise and investigate loneliness among older adults and whether these studies incorporate multidisciplinary perspectives in both theoretical frameworks and intervention strategies. The review focuses on literature published in the pre-COVID and COVID-19 periods in order to explore potential shifts in research approaches during the pandemic. The post-pandemic phase was not included because the objective is to capture the immediate impact of the COVID-19 crisis on research orientations, while longer-term developments are still evolving and remain difficult to assess systematically.

The review addresses the following research questions:

1. How is loneliness among older adults approached within biological, clinical, and psychosocial studies?
2. How frequently is loneliness examined from a multidisciplinary perspective?
3. Did the COVID-19 pandemic influence the adoption of multidisciplinary approaches in loneliness research on community-dwelling older adults?

Throughout this article, the term *multidisciplinarity* is used as an overarching concept to describe the coexistence and interaction of perspectives from different disciplinary fields.

## 2 Methods

### 2.1 Study design

This study is a systematic literature review conducted in accordance with the PRISMA 2020 Statement (42). To ensure robustness, two researchers were assigned to each thematic/disciplinary area: biological (MB, RG), clinical (GF, IB), and socio-psychological (GC, SS), with a supervisor (GL). Regular coordination meetings addressed uncertainties and monitored research phases, ensuring consistency throughout the process.

### 2.2 Search strategy and study selection

Between February 2023 and March 2024, the research team conducted a comprehensive search of major academic databases (PubMed, Scopus, Web of Science, Embase, and EBSCO), employing predefined and domain-specific keywords, as detailed in Table 1. The search strategy combined free-text keywords with controlled vocabulary terms (e.g., MeSH in PubMed and EMTREE in Embase), using Boolean operators (AND, OR) to structure and refine the search queries across databases. This approach is commonly recommended in systematic reviews in order to maximise the retrieval of relevant studies while maintaining an appropriate balance between sensitivity and specificity in the search process (43).

**Table 1:** Keywords by research area.

Research area	Keywords
Biological	Pubmed, Scopus, Web of Science: (older adults OR elderly OR older people) AND loneliness AND (cortisol OR inflammation) Embase: (older adults OR elderly OR older people) AND loneliness AND (cortisol OR (inflammation AND biological marker))
Clinical	(older adults OR elderly OR older people) AND loneliness AND (disability OR multimorbidity OR co-morbidity OR comorbidity) AND community dwelling
Socio-psychological	(older adults OR elderly OR older people) AND loneliness AND community dwelling

In line with the study’s objective to adopt a multidisciplinary perspective, keywords were carefully selected for each research domain in order to capture the conceptual nuances of loneliness within the respective research contexts. At the same time, the selection of keywords was designed to ensure that the search strategy remained sufficiently inclusive, allowing the identification of relevant studies without imposing overly restrictive criteria that might lead to excessive filtering of potentially pertinent literature.

For instance, the term “cortisol” was employed within the biological literature, as cortisol levels are frequently investigated as physiological indicators of loneliness. Similarly, keywords such as “comorbidity” and “multimorbidity” were used to identify relevant clinical studies. Given the central role of loneliness in the socio-psychological literature, additional domain-specific terms were not considered necessary. Moreover, the selection of keywords for each area was deliberately limited to those strictly necessary to orient the search towards the core theme of loneliness. Broader terms related to the ageing process (e.g., physical limitations) were excluded, as they were considered implicitly encompassed by the term “older people.”

We selected papers published between 2016 and 2023 and grouped them into two periods: the pre-COVID-19 phase (2016–2019) and the COVID-19 pandemic phase (2020–2023). The starting year (2016) was chosen to focus on the most recent developments in loneliness research among older adults and the growing scientific interest in this topic in the years immediately preceding the pandemic. The year 2020 was considered a turning point due to the global impact of COVID-19. This temporal categorisation was defined *a priori* to explore potential changes in the literature associated with the pandemic context. The search was limited to publications up to 2023 in order to include studies produced while the pandemic crisis was still being actively managed. The selected intervals were intended to provide a balanced comparison between the pre-pandemic and pandemic periods. Each pair of researchers reviewed the studies based on title and abstract to assess relevance within their area of interest. Studies were included if they met the following inclusion criteria: a) being focused on loneliness; b) being research articles; c) involving individuals aged 65 or older; d) targeting community-dwelling populations; e) examined the biological correlates of loneliness related to stress or immune pathways, particularly the hypothalamic–pituitary–adrenal (HPA) axis and inflammatory processes; and f) being written in English. Literature reviews, methodological papers, grey literature, and book chapters were excluded, as well as papers in languages other than English. At the end of the selection process, 131 studies met the eligibility criteria and were included in the review. No additional references were found through manual searching or in the references of the included articles.

### 2.3 Data collection, extraction and analysis

The research team established data collection procedures, units of analysis, variables, and categories to ensure consistency across all included studies. Based on these elements, a structured data extraction table was developed (Table 2). Two main units of analysis were identified: (1) study characteristics and (2) content-related variables. The first unit includes general information such as author, year of publication, methodology, and sample size, providing a descriptive overview of the existing literature. The second unit focuses on the core themes addressed in the review, namely loneliness and the presence of multidisciplinary approaches.

**Table 2:** Units of analysis, variables and categories.

Units of analysis	Main variables	Modalities
Study characteristics		
General information	Author(s)	Reference
	Year of publication	Before 2020 After 2020
Methodology	Methods	Qualitative Quantitative Mixed method
	Type of study	Cross-sectional Longitudinal Observational Survey Randomised Controlled Trials Pre-post study Secondary data study
Sample	Sample size	≤ 50 51–100 101–200 201–300 ≥ 301
	Age group	Over 65 Over 80
	Sex	Mono sex target Both sexes
	Health conditions considered	Yes No
Contents of studies		
Keywords	List of keywords	-
	Presence of “loneliness” among keywords	Yes No
Outcome	Loneliness as primary or secondary outcome	Primary Secondary
Multidisciplinary approach	Multidisciplinary approach in the methods	Yes No
	Multidisciplinary approach in the discussion	Yes No
Multidisciplinary considerations	Considerations/recommendations on the integration of different disciplines	Yes No

Specifically, the following information was collected: (1) keywords reported in each manuscript; (2) whether loneliness was investigated as a primary or secondary outcome; (3) the tools used to assess loneliness (e.g., validated scales); and (4) the adoption of multidisciplinary approaches, either through the use of methods originating from different disciplines (e.g., the combination of psychometric instruments and biological markers) or through explicit discussion of the need for multidisciplinary perspectives in the interpretation of results.

The variables related to loneliness and multidisciplinary approaches were designed to identify both their presence and their relevance within the selected studies. In particular, regarding loneliness as an outcome, we examined whether the term “loneliness” appeared among the article’s keywords and whether specific instruments were used to measure it (e.g., validated psychometric scales). To identify the presence of multidisciplinary perspectives, we assessed whether contributions from different disciplinary domains were integrated within the study design, methodological approach, or interpretation of findings.

The categorisation of variables followed principles of qualitative categorisation described by Guest et al. (44), with the aim of: (i) identifying the presence or absence of relevant variables in each study; (ii) distinguishing different response modalities for the selected variables; and (iii) facilitating their systematic comparison and interpretation. A content analysis approach was applied in order to collect comparable information across all studies (45). To ensure consistency in the data extraction process, the first author conducted a final review of all coded variables and discussed any uncertainties or duplications with the research team.

A frequency distribution was subsequently performed to support the descriptive statistical analysis, providing an overview of the main variables and their distribution across the included studies.

Given the substantial heterogeneity of study designs across the three research domains (biological, clinical, and socio-psychological), and the exploratory aim of mapping multidisciplinary perspectives rather than evaluating intervention effectiveness, a formal quality appraisal using standardised tools was not conducted. However, the methodological characteristics of each study were carefully examined during the screening and data extraction phases in order to ensure that all included papers met the eligibility criteria and provided sufficient information to address the objectives of the review. This methodological choice is consistent with the exploratory aim of the review, which was to map multidisciplinary perspectives across heterogeneous bodies of literature rather than to evaluate intervention effectiveness or estimate pooled effects.

## 3 Results

### 3.1 Selection process

**Figure 1:**
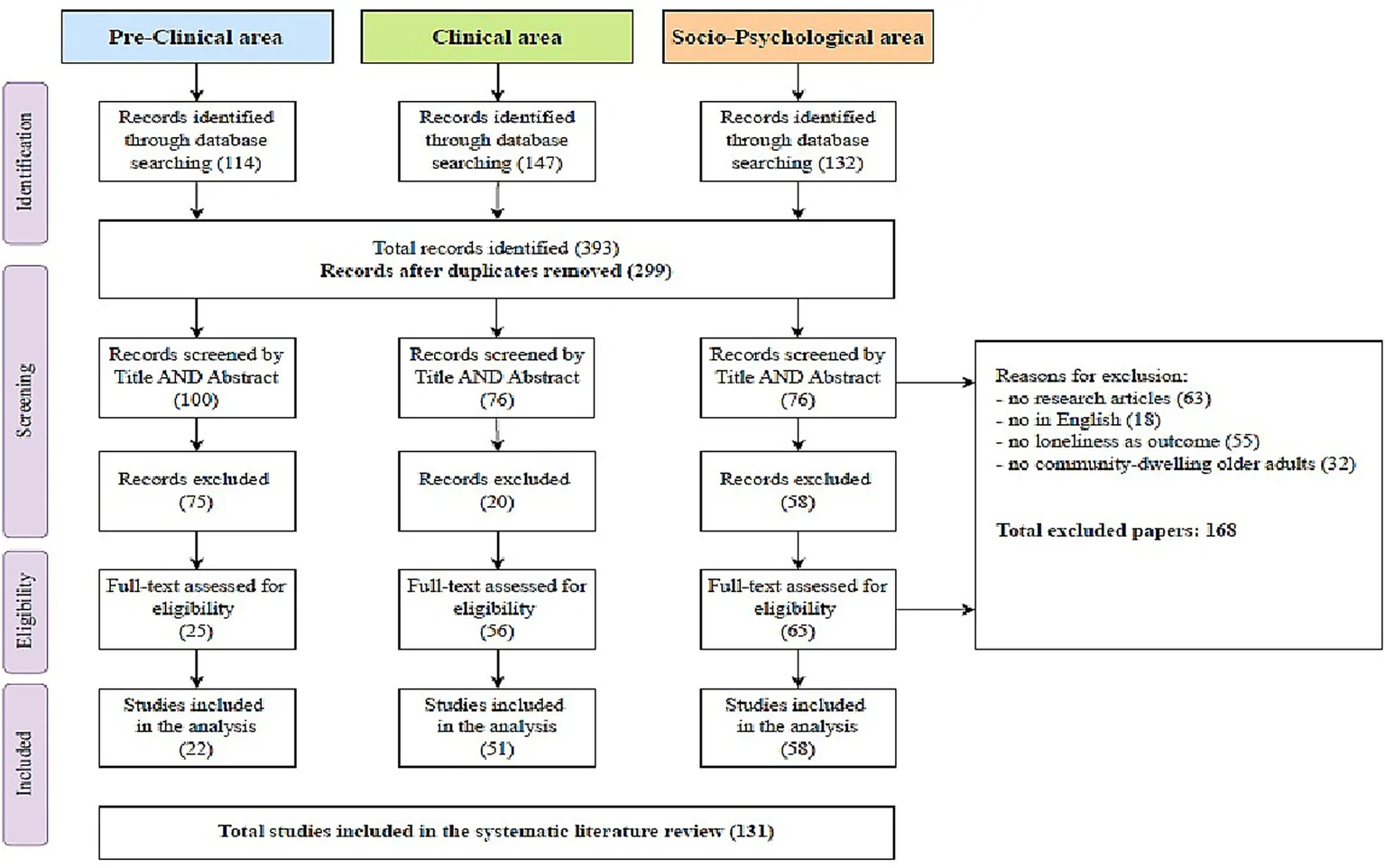
Flowchart of review according to the PRISMA 2020 checklist.

We initially identified 393 articles: 114 in the biological domain (BL), 147 in the clinical domain (CL), and 132 in the socio-psychological domain (SP). After duplicate records were removed, 299 articles remained for screening. A further 168 records were excluded following title, abstract, and full-text screening. Ultimately, 131 studies met the eligibility criteria and were included in the review, comprising 22 biological studies (17%), 51 clinical studies (39%), and 58 socio-psychological studies (44%). Figure 1 presents the PRISMA flow diagram of the study selection process, while Annex 1 provides the complete list of the included studies.

### 3.2 Characteristics of selected studies

This section provides an overview of the methodological and descriptive characteristics of the included studies across the three research domains. Out of the 131 studies included in the review, 55 were published between 2016 and 2019 and 76 between 2020 and 2023, indicating an increase in publications during the COVID-19 period. The most marked increase was observed in the clinical domain, where the number of studies rose by 55% compared with the pre-pandemic period. More modest increases were observed in the socio-psychological (+32%) and biological (+20%) domains. Table 3 summarises the main methodological and descriptive characteristics of the included studies by research domain.

**Table 3:** Characteristics of selected papers by disciplinary area.

Units of analysis / modalities	Biological (*N* = 22) abs. value	Clinical (*N* = 51) abs. value	Socio-psychological (*N* = 58) abs. value	Total (*N* = 131) abs. value
Publication Year				
2016–2019	10	20	25	55
2020–2023	12	31	33	76
Methods				
Quantitative	22	24	43	89
Qualitative	0	6	11	17
Mixed-method	0	21	4	25
Type of study				
Cross-sectional	9	33	17	59
Observational	6	6	13	25
Longitudinal	6	10	5	21
RCTs	0	1	11	12
Survey	1	1	7	9
Pre-post	0	0	3	3
Secondary data	0	0	2	2
Sample size				
<50	0	0	10	10
Between 50 and 100	4	1	11	16
Between 101 and 300	4	11	7	22
>300	14	39	30	83
Age of participants				
65-80-year-old	22	40	48	110
>80	0	11	9	20
Not Available	0	0	1	1
Health condition as inclusion criteria				
Yes	20	42	21	83
No	2	9	37	48
Sex				
Single sex	0	1	1	2
Both sex	22	50	56	128
Not Available	0	0	1	1
Geographical areas*				
Africa	0	1	0	1
Asia	0	5	2	7
Europe	10	22	24	56
Far East	2	4	11	17
Intercontinental	0	0	1	1
Latin America	0	2	0	2
Middle East	0	1	4	5
North America	10	15	10	35
Oceania	0	1	6	7

*Africa: Ghana. Asia: Bangladesh; India, Malaysia, Indonesia; Europe: Belgium, Denmark, Netherlands, Finland, Germany, Ireland, Italy, Spain, Sweden, UK; Far East: China, Hong-Kong, Singapore, Taiwan, Japan; Intercontinental: Canada and Norway; Latin America: Brazil and Mexico; Middle East: Israel, Iran, Turkey; North America: Canada and U. S.; Oceania: Australia and New Zealand.

Regarding methodological approaches, most studies employed quantitative methods (68%), while 13% adopted qualitative methodologies and 19% applied mixed-method approaches. All biological studies used quantitative designs, whereas clinical and socio-psychological studies displayed greater methodological diversity. In particular, mixed-method approaches were relatively frequent among clinical studies (21 studies, 41%), while socio-psychological research was predominantly quantitative (43 studies, 74%).

In terms of study design, most investigations were cross-sectional (59 studies, 45%), observational (25 studies, 19%), or longitudinal (21 studies, 16%). Nearly all biological and clinical studies fell within these categories. In contrast, socio-psychological research also included a notable number of randomised controlled trials (RCTs) and survey-based studies, while pre-post designs and secondary data analyses were less common.

With regard to sample size, most studies (83, or 63.5%) involved more than 300 participants. However, socio-psychological studies were more frequently based on smaller samples, with approximately one in five including fewer than 100 participants.

Concerning participants’ age, in 84% of the studies the target population consisted of adults aged between 65 and 80 years. The remaining studies focused on individuals aged over 80 years and were equally distributed between clinical and socio-psychological investigations, whereas none of the biological studies addressed this age group. Almost all studies (98%) included participants of both sexes; only two studies focused on single-sex samples in the clinical and socio-psychological areas.

Health status was reported as an inclusion criterion in nearly two-thirds of the studies (63%), although with substantial differences across research domains. This criterion was considered in almost all biological studies (91%) and in the majority of clinical studies (82%), whereas it was reported less frequently in socio-psychological research (36%).

Regarding geographical distribution, most studies were conducted in Europe (43%), followed by North America (27%) and East Asia (13%), while Asia and Oceania each accounted for approximately 5% of the total.

### 3.3 Loneliness as a keyword and outcome

This section examines how loneliness was conceptualised within the included studies by analysing its role as a keyword, its position as a primary or secondary outcome, and the measurement tools adopted. The analysis of whether the term *loneliness* was included among article keywords and considered as a primary or secondary outcome shows that it appeared explicitly in the keyword list in 47% of the studies and was absent in 37% of the articles. In the remaining cases (approximately 15%), the consulted articles did not report a keyword list.

Comparing the three research domains, the presence of the term *loneliness* among keywords was considerably higher in biological studies (16 out of 22) and socio-psychological studies (32 out of 58), while it was less frequent in clinical research (14 out of 51). As shown in Table 4, the inclusion of loneliness among article keywords does not necessarily correspond to whether loneliness is investigated as a primary or secondary outcome. Moreover, the distribution of studies before and during the COVID-19 pandemic remains relatively balanced in terms of loneliness being analysed as a primary or secondary outcome.

**Table 4:** Loneliness as primary or secondary outcome, by loneliness as keyword, time of publication and research area.

Loneliness as outcome	Total	Loneliness as keyword			Time of publication		Research area		
		Yes	No	N. A.	2016–2019	2020–2023	BL	CL	SP
Primary	61	40	16	5	26	35	11	10	40
Secondary	70	22	33	15	29	40	11	41	17
Not available	1	0	0	1	0	1	0	0	1
Total	131	62	49	20	55	76	22	51	58

Overall, loneliness was examined as a primary outcome in 61 studies and as a secondary outcome in the remaining cases. This trend was particularly evident in socio-psychological studies (62%) and in biological investigations (50%).

**Figure 2:**
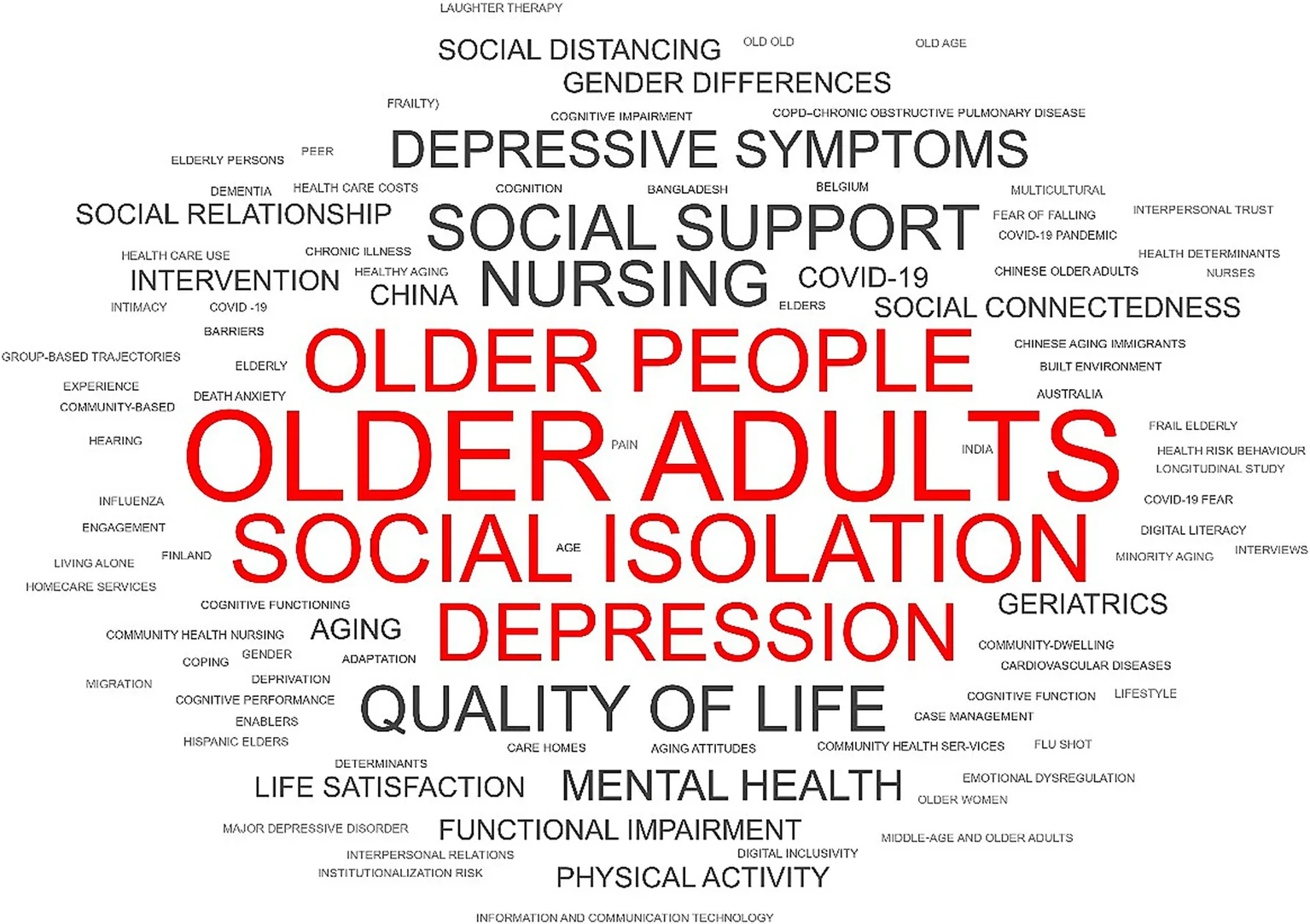
Word cloud of keywords associated with loneliness.

Figure 2 complements this analysis through a word cloud illustrating the frequency of keywords associated with loneliness when the term was included among article keywords. The cloud excludes the terms *older adults* and *older people*, which were used as part of the search strategy. The most frequent associated keywords were *social isolation* (11 occurrences), *depression* (8), and *cortisol* (7). Other relatively frequent terms included *quality of life*, *nursing*, and *social support* (five occurrences each). Less frequent keywords related to the physical condition of older adults included *functional impairment* and *physical activity* (two occurrences each). The terms *social connectedness* and *social distancing* also appeared twice. The association of loneliness with keywords such as *depression*, *nursing*, and mental health–related concepts highlights the strong health-related dimension through which loneliness is often approached in the literature.

To further examine the role of loneliness in the selected studies, we also analysed the tools used to measure it. The type and quality of the instrument adopted may significantly influence both the reliability of the analysis and the relevance attributed to loneliness within the study. Approximately 75% of the studies assessed loneliness using a specialised validated scale. A smaller proportion (11%) relied on a single-item question, such as “How often did you feel lonely in the past week?” (59). In addition, 7% of the studies used non-validated scales, while another 7% did not include a specific scale or question to measure loneliness. As shown in Table 5, validated scales were widely used across all three research domains. However, some clinical studies relied on single-item measures or non-validated tools. Socio-psychological studies employed a wider range of measurement approaches, including qualitative and non-standardised instruments, reflecting methodological choices associated with qualitative research designs (46).

**Table 5:** Assessment tools for loneliness by research area.

Assessment tools used for measuring loneliness (N)	Biological (BL) (22)	Clinical (CL) (51)	Socio-psychological (SP) (58)	Total (131)
Validated scales	21	35	42	98
Single-item question	1	10	4	15
Non-validated scales	0	6	3	9
No specific measure	0	0	9	9
	22	51	58	131

### 3.4 Multidisciplinary research

One of the main objectives of this review was to examine the extent to which multidisciplinary perspectives are incorporated into loneliness research among community-dwelling older adults. The combined analysis of loneliness and multidisciplinary variables provides insights into how these two dimensions intersect within the literature on community-dwelling older adults. Tables 6, 7 present the distribution of different types of multidisciplinary approaches when loneliness is considered, respectively, as a primary and as a secondary outcome.

**Table 6:** Level of multidisciplinary approach by time of publication and research area.

Multidisciplinarity	Total	Time of publication		Research area		
	N	2016–2019	2020–2023	BL	CL	SP
Missing	64	26	38	0	18	46
Adopted as a methodological approach	26	11	15	21	1	4
Adopted in the discussion of findings	14	8	6	0	13	1
Full (methods + discussion)	27	10	17	1	19	7
Total	131	55	76	22	51	58

**Table 7:** Multidisciplinarity by loneliness as keyword and as a primary or secondary outcome.

Multidisciplinarity	Total	Loneliness in the keywords			Loneliness as outcome	
	N	Yes	No	N. A.	Primary	Secondary
Missing	64	33	24	7	38	26
Adopted as a methodological approach	26	16	8	2	1	25
Adopted in the discussion of findings	14	4	5	5	4	10
Full (methods + discussion)	27	9	12	6	7	20
Total	131	62	49	20	50	81

The data reported in Table 6 show that 20% of the studies applied a multidisciplinary methodological approach in conducting their research, while 10% discussed their findings from a multidisciplinary perspective. A further 21% adopted a fully multidisciplinary approach, integrating multidisciplinarity both in the methodological design and in the discussion of results. However, nearly half of the articles (49%) adopted a sector-specific approach, with no explicit reference to multidisciplinary perspectives in the conceptual framework, methodology, or interpretation of findings.

Among the 27 articles adopting a fully multidisciplinary approach, 10 were published before 2020 and 17 during the COVID-19 period. Similarly, 26 studies employed multidisciplinary methods, 11 of which were published before 2020. Fourteen articles adopted a multidisciplinary perspective only in the discussion of findings, eight of them published before the pandemic.

The analysis by research domain reveals distinct patterns. All biological studies employed multidisciplinary methods, although only one explicitly incorporated a multidisciplinary perspective in the discussion of results. Among clinical studies, 18 (35% of 51) did not adopt any multidisciplinary perspective, 13 (25%) included a multidisciplinary discussion of findings, and 19 (37%) applied a fully multidisciplinary approach combining methodological and interpretative perspectives. In contrast, the majority of socio-psychological studies (46 out of 58) did not explicitly adopt a multidisciplinary approach; only four applied multidisciplinary methods and seven adopted a broader multidisciplinary research framework.

Finally, examining the relationship between multidisciplinary approaches and the role of loneliness as a research outcome (Table 7) shows that multidisciplinary perspectives are more frequently adopted when loneliness is treated as a secondary outcome. In these cases (55 studies, 42% of the total), multidisciplinary approaches appear mainly in methodological design (25 studies), followed by fully multidisciplinary approaches (20 studies) and multidisciplinary discussions of results (10 studies). Conversely, when loneliness is considered the primary research outcome, multidisciplinary approaches are less common, with only 12 studies adopting them either in methodological design or in the interpretation of findings. Overall, these findings indicate that multidisciplinary approaches are more frequently adopted when loneliness is examined in relation to broader health conditions than when it constitutes the primary focus of the research.

## 4 Discussion

### 4.1 Innovative aspects of this study

This review aimed to examine the extent to which research on loneliness among community-dwelling older adults adopts a multidisciplinary perspective by comparing biological, clinical, and socio-psychological research before and during the COVID-19 pandemic. To our knowledge, this is the first systematic literature review examining loneliness among community-dwelling older adults from a multidisciplinary perspective. By analysing 131 studies across three research domains—biological (BL), clinical (CL), and socio-psychological (SP)—this review explored how loneliness is conceptualised and investigated, and to what extent multidisciplinary approaches are applied in research design and interpretation.

The findings show that loneliness among older adults has been widely investigated across the three disciplinary domains considered. However, both before and during the COVID-19 pandemic, research has tended to focus primarily on the health consequences of loneliness rather than on its broader social and psychological determinants. At the same time, the number of studies adopting multidisciplinary perspectives increased during the pandemic period.

The findings suggest that the COVID-19 pandemic may have contributed to greater interaction between research domains. The public health emergency highlighted the complex interaction between social isolation, psychological distress, and physical health risks among older adults, making it increasingly difficult to address loneliness from a single disciplinary perspective. As a consequence, the pandemic context appears to have stimulated a broader recognition of loneliness as a multidimensional phenomenon requiring contributions from different fields of research. Although it is still too early to determine whether this shift represents a long-term transformation of research practices, the increase in multidisciplinary approaches observed during the pandemic years suggests an important change in the scientific framing of loneliness in older populations. Nevertheless, despite this positive trend, disciplinary fragmentation remained the predominant pattern across the literature. These findings indicate that, although multidisciplinary research is increasing, the integration of biological, clinical, and socio-psychological perspectives is still evolving.

The growing attention to loneliness can also be explained by the increasing evidence linking this condition to a wide range of health outcomes. Previous studies have documented associations between loneliness and mental and physical health conditions, including depression, cardiovascular diseases, and cognitive decline (47). Other research has explored neurobiological mechanisms associated with loneliness (60) as well as the effectiveness of interventions aimed at reducing loneliness among older adults (48–50). Although loneliness has long been a central topic in the social and psychological sciences, it has attracted increasing interest in biological and clinical research more recently, particularly during the COVID-19 pandemic, when social distancing measures highlighted its potential implications for health (51).

The multifaceted nature of loneliness (4) is reflected in the diversity of methodological approaches used to study it. Both qualitative and quantitative designs are employed, and a range of instruments are used to measure loneliness, most commonly validated scales. Recent literature has emphasised the importance of selecting measurement tools that are appropriate to the study objectives and context (52, 53). Our findings suggest that methodological choices often reflect disciplinary traditions rather than the specific characteristics of the multifaceted phenomenon of loneliness in its entirety. In several cases, the description of sample characteristics was relatively limited, confirming previous observations that research on loneliness sometimes relies on broadly defined population profiles rather than highly differentiated samples (54).

### 4.2 Disciplinary perspectives on loneliness

The analysis highlights important differences in how loneliness is conceptualised across the three research domains considered in this review.

In the biological literature, loneliness is primarily investigated in relation to physiological mechanisms such as stress responses, inflammatory processes, and immune functioning. Although interest in this topic increased during the pandemic, biological studies tend to frame loneliness mainly as a factor influencing biological pathways and health outcomes, with limited attention to the broader social and psychological determinants of the phenomenon. Interestingly, while biological studies frequently integrate biological and clinical perspectives, the inclusion of socio-cultural dimensions remains relatively limited.

A similar pattern emerges in the clinical literature. In this field, loneliness is often analysed in relation to depression and other mental health conditions. While these associations are well documented, loneliness should not be interpreted simply as an early stage of depression. Rather, it represents a distinct condition with specific determinants and consequences. Recognising this distinction is important for clinical practice, as loneliness may both contribute to and exacerbate certain psychological and physical health conditions in older adults.

In contrast, socio-psychological studies tend to emphasise the relationship between loneliness and social isolation, highlighting the importance of social support, social participation, and community engagement in preventing or mitigating loneliness. However, these studies rarely explore the biological or clinical implications of loneliness. Interpreting such implications often requires expertise that lies outside the traditional scope of social or behavioural research. This highlights the challenges involved in establishing multidisciplinary research teams, which may face barriers related to training, professional identity, communication, and institutional structures (55, 56).

Overall, the results suggest that research on loneliness among older adults still tends to develop within relatively distinct disciplinary boundaries. Multidisciplinary approaches are present but often involve collaboration among closely related disciplines rather than fully integrated perspectives. Some scholars have suggested that multidisciplinarity may represent an intermediate step towards transdisciplinary approaches, particularly in the field of ageing research (57). In this context, strengthening multidisciplinary research may provide a solid foundation for the future development of more integrated interdisciplinary and transdisciplinary approaches. Given the complex interactions between biological, clinical, and socio-psychological determinants of loneliness, progressively expanding collaboration across disciplinary boundaries may support the development of more comprehensive strategies to understand and address loneliness among older adults.

### 4.3 Implications for research and policy

The findings of this review suggest several implications for future research and policy development. First, greater collaboration between biological, clinical, and socio-psychological research domains may contribute to a more comprehensive understanding of loneliness among older adults. Developing multidisciplinary research designs that integrate physiological, psychological, and social indicators could help clarify the complex interactions underlying loneliness and its health consequences.

Second, recognising loneliness as a multifactorial condition highlights the importance of integrated policy responses. Strategies aimed at addressing loneliness among community-dwelling older adults may benefit from coordinated interventions that combine healthcare services, social care systems, and community-based initiatives. Such approaches could support more comprehensive prevention and intervention strategies capable of addressing both the social and health-related determinants of loneliness.

### 4.4 Study limitations

Several limitations should be considered when interpreting the findings of this review. First, the heterogeneity of the included studies did not allow the use of a meta-analytic approach to synthesise results across research domains. Second, the use of frequency distributions as the primary analytical method allowed the identification of general trends but limited the possibility of conducting more in-depth analyses within each disciplinary area. Third, restricting the search to articles published in English may have introduced language bias, although English remains the dominant language in this field of research. Fourth, given the substantial heterogeneity of study designs across the three research domains, a formal quality appraisal using standardised tools (such as CASP or MMAT) was not conducted. The application of these instruments requires uniform methodological criteria, which are difficult to apply across highly diverse study designs (58). For this reason, future research may benefit from conducting separate, domain-specific reviews applying quality assessment tools appropriate to each disciplinary field. Finally, this review focused on three research domains—biological, clinical, and socio-psychological—while other relevant perspectives, such as rehabilitation sciences or economic research, were not included. Expanding the disciplinary scope of future reviews may provide a more comprehensive understanding of loneliness among older adults. Despite these limitations, this review represents, to our knowledge, the first attempt to investigate the extent to which multidisciplinary approaches are adopted in research on loneliness among community-dwelling older adults, highlighting loneliness as a multifaceted phenomenon encompassing biological, clinical, and socio-psychological dimensions.

## 5 Conclusion

This study examined loneliness among community-dwelling older adults from a multidisciplinary perspective by analysing biological, clinical, and socio-psychological research. The analysis of 131 studies shows that loneliness has been widely investigated across these domains, yet the adoption of fully integrated multidisciplinary approaches remains limited.

Compared with the pre-pandemic period, the findings suggest that the COVID-19 pandemic was associated with increased attention to multidisciplinary perspectives in loneliness research, highlighting the complex interactions between social, psychological, and health-related factors affecting older adults.

These findings underline the need to strengthen collaboration between social, psychological, biomedical, and health-related disciplines in order to better understand and address loneliness as a multifaceted condition affecting older people’s health and well-being.

Future research could benefit from more advanced analytical approaches, including meta-analyses or qualitative content analyses focusing on specific disciplinary areas. In addition, further reviews of studies published after 2023 may help assess whether the increased attention to multidisciplinary approaches observed during the COVID-19 period represents a long-term development in loneliness research.

## Data Availability

Data are available under request to the corresponding author.
