# Correction: Loneliness among community-dwelling older adults across biological, clinical and psychosocial domains before and during the COVID-19 pandemic: a systematic review

**DOI:** 10.3389/fpubh.2026.1963451

**Published:** 2026-09-02

**Authors:** Georgia Casanova, Giorgia Fattorini, Sara Santini, Marta Balietti, Ilaria Barboni, Robertina Giacconi, Giovanni Lamura

**Affiliations:** 1 Centre for Socio-Economic Research on Aging, IRCCS INRCA, Ancona, Italy; 2 Department of Experimental and Clinical Medicine, Section of Neuroscience and Cell Biology, Università Politecnica delle Marche, Ancona, Italy; 3 Center for Neurobiology of Aging, IRCCS INRCA, Ancona, Italy; 4 Clinical Unit of Physical Rehabilitation, IRCCS INRCA, Ancona, Italy; 5 Advanced Technology Center for Aging Research and Geriatric Mouse Clinic, IRCCS INRCA, Ancona, Italy

**Keywords:** biological clinical and psychosocial domains people, community-dwelling, COVID-19, loneliness, multidisciplinary research, older adults, systematic review

Marta Balietti was incorrectly affiliated with affiliation 2 “Department of Experimental and Clinical Medicine, Section of Neuroscience and Cell Biology, Università Politecnica delle Marche, Ancona, Italy”. This author should have affiliation 3 “Center for Neurobiology of Aging, IRCCS INRCA, Ancona, Italy”.

There was an error in affiliation 4, “INRCA IRCCS” should be “IRCCS INRCA”.

Ilaria Barboni was affiliated with affiliations 4 and 5. This author should only have affiliation 4 “Clinical Unit of Physical Rehabilitation, IRCCS INRCA, Ancona, Italy”. Affiliation 5 “Advanced Technology Center for Aging Research and Geriatric Mouse Clinic, IRCCS INRCA, Ancona, Italy” has been removed for this author.

Affiliation “Department of Clinical and Molecular Sciences, Università Politecnica delle Marche, Ancona, Italy” was erroneously included in the paper. This affiliation has now been removed for author Robertina Giacconi.

Robertina Giacconi should be affiliated with affiliation 5 “Advanced Technology Center for Aging Research and Geriatric Mouse Clinic, IRCCS INRCA, Ancona, Italy”.

The original version of this article has been updated.

